# Association between relative fat mass and cardiovascular disease: a cross sectional study based on NHANES

**DOI:** 10.3389/fcvm.2025.1590979

**Published:** 2025-06-24

**Authors:** Bin Zhang, Changguo Zhu, Lanhua Ma, Bai Shen, Gaoxing Zhang

**Affiliations:** ^1^ Department of Hypertension and Vascular Disease, The First Affiliated Hospital, Sun Yat-sen University, Guangzhou, Guangdong, China; ^2^ Department of Cardiovascular Disease and Clinical Experimental Center, Jiangmen Central Hospital, Jiangmen, Guangdong, China; ^3^ Center for Sleep and Circadian Medicine, The Affiliated Brain Hospital of Guangzhou Medical University, Guangzhou, Guangdong, China; ^4^ Department of Respiratory, The General Hospital of the Third Division of Xinjiang Production and Construction Corps, Tumxuk, Xinjiang, China; ^5^ Department of Cardiovascular Medicine, The Affiliated Dongguan Songshan Lake Central Hospital, Guangdong Medical University, Dongguang, Guangdong, China

**Keywords:** relative fat mass, cardiovascular disease, obesity, NHANES, waist-to-height ratio

## Abstract

**Background:**

Cardiovascular disease (CVD) represent a primary factor contributing to death worldwide. Conventional indicators of obesity, waist circumference, waist-to-hip ratio, body mass index (BMI), have limitations in differentiating between fat and muscle mass. Relative fat mass (RFM), a novel metric based on waist to height ratio, has been proposed as a more accurate measure of total body fat percentage. This research examines the relationship between RFM and CVD, utilizing data sourced from the National Health and Nutrition Examination Survey (NHANES).

**Methods:**

This cross sectional study utilized data sourced from the National Health and Nutrition Examination Survey (NHANES) from 1999 to 2018. Participants with unavailable data on waist circumference, height, CVD complications, or total cholesterol were excluded. A total of 45,000 participants were included and divided into quartiles based on RFM values. Multivariate logistic regression models were used to assess the association between RFM and CVD. Moreover, analyses including subgroup evaluations, smooth curve modeling, and testing for interactions were conducted.

**Results:**

The prevalence of CVD was 10.42% (4,691) among the 45,000 participants. Fully adjusted models showed a significant positive association between RFM and CVD (OR = 1.04; 95% CI = 1.03–1.05; *P* < 0.001). Participants in the highest RFM quartile(Q4) had a 2.11 fold increased risk of CVD compared to those in the lowest quartile (OR = 2.11; 95% CI = 1.76–2.53; *P* < 0.001). Subgroup analyses indicated that the association was stronger in individuals aged <60 years, nonHispanic Whites, and those with BMI < 30 kg/m^2^.

**Conclusions:**

Elevated RFM is associated with an higher proportion of patients with CVD, suggesting that RFM may be a valuable indicator for CVD prevention and management. Future prospective studies are warranted to further explore the causal relationship between RFM and CVD.

## Introduction

Cardiovascular disease (CVD) are among the primary drivers of illness and death (1), with the most common types being heart attacks and strokes (2). The burden of CVD continues to escalate, driven by urbanization, sedentary lifestyles, and an aging population (3). Obesity, another growing health crisis, is intricately linked to CVD (4), affecting, along with overweight, over a third of the world's population today, with this trend affecting both high- and low-income countries (5). Obesity contributes to metabolic dysregulation and inflammation, increasing the risk of hypertension, type2 diabetes, and atherosclerosis (6, 7). Traditional obesity metrics like BMI and waist circumference (WC) fail to distinguish between fat and muscle or accurately reflect body fat distribution, limiting their utility in assessing cardiovascular risk (8, 9).

In response, the Relative Fat Mass (RFM) metric has surfaced as an innovative method for assessing body fat levels. Derived from an algorithm that compares waist circumference to height, RFM has been corroborated through dual-energy x-ray absorptiometry (DXA) and has been found to provide a more precise estimation of overall body fat percentage than BMI (10). RFM has demonstrated strong associations with hypertension (11), type2 diabetes (12), and stroke (13). Highlighting its potential as a cardiometabolic risk indicator. Nonetheless, the connection between RFM and cardiovascular disease has not been thoroughly investigated.

This study aims to fill this gap by investigating the association between RFM and CVD. Our findings may provide valuable insights into the role of RFM in cardiovascular risk assessment and contribute to the development of targeted prevention strategies for high-risk populations.

## Materials and methods

### Study population

The information utilized originated from the National Health and Nutrition Examination Survey (NHANES) that were carried out by the National Center for Health Statistics (NCHS) over the period from 1999 to 2018. Access to all pertinent data is available at: https://www.cdc.gov/nchs/nhanes/. The NHANES data set includes various health indicators such as demographic characteristics, physical examination results and laboratory findings. The exclusion criteria for this study included: (1) missing or unavailable data on waist circumference data. (2) Missing or unavailable height data. (3) Missing or unavailable CVD complications data. (4) Missing or unavailable total cholesterol.

### Variables

#### Cardiovascular disease

CVD was established using self-reported information gathered from the Medical Condition Questionnaire, which inquired whether participants had ever received a diagnosis of coronary heart disease, had experienced a stroke, angina, suffered a heart attack, or been diagnosed with congestive heart failure.

#### RFM

RFM was calculated using the formula: RFM = 64 − (20 × Height/Waist Circumference) + (12 × Gender), where gender was coded as 1 for females and 0 for males (10, 13).

### Covariates

Covariates included age, gender, race, education level, marital status, family poverty income ratio (PIR), smoking and drinking status, diabetes, hypertension, and total cholesterol (TC).

### Statistical analysis

R Statistical Software (Version 4.2.2, http://www.R-project.org, The R Foundation) and the Free Statistics analysis platform (Version 1.9.2, Beijing, China, http://www.clinicalscientists.cn/freestatistics) were used for analysis. Statistical significance was evaluated using a two-tailed test with a threshold of *p* of <0.05. Continuous variables were described as mean ± standard deviation (SD), and categorical variables as percentages.

To assess the relationship between RFM and CVD, we employed multivariate logistic regression analyses to estimate the odds ratios (OR) and corresponding 95% confidence intervals (CI). The analysis included three distinct models: Model 1 was unadjusted, providing a baseline assessment of the association. Model 2 adjusted for key demographic factors, including sex, age, and ethnicity. Model 3 incorporated additional adjustments for marital status, PIR, education level, smoking status, alcohol consumption, hypertension, diabetes, and TC (13).

To evaluate the dose-response relationship between RFM and CVD, we utilized a restricted cubic spline (RCS) regression model with four knots positioned at the 25th, 50th, 75th and 100th percentiles of RFM. This approach allowed us to assess linearity and explore the relationship after adjusting for the covariates in Model 3.

Interaction and subgroup analyses were also conducted through logistic regression models, differentiating participants based on factors such as age, gender, ethnicity, body mass index, smoking habits, alcohol intake, hypertension, and diabetes (13). These evaluations were instrumental in uncovering possible influencers on the effect and in examining whether the association remained consistent across various demographic segments.

Sensitivity analyses were conducted to ensure the robustness of our results. First, we excluded participants with missing data on RFM or CVD status to evaluate whether the results were sensitive to Non-Hispanic White (race 1, details in Supplementary Material).

## Results

### Participant flow and study population

**Figure 1:**
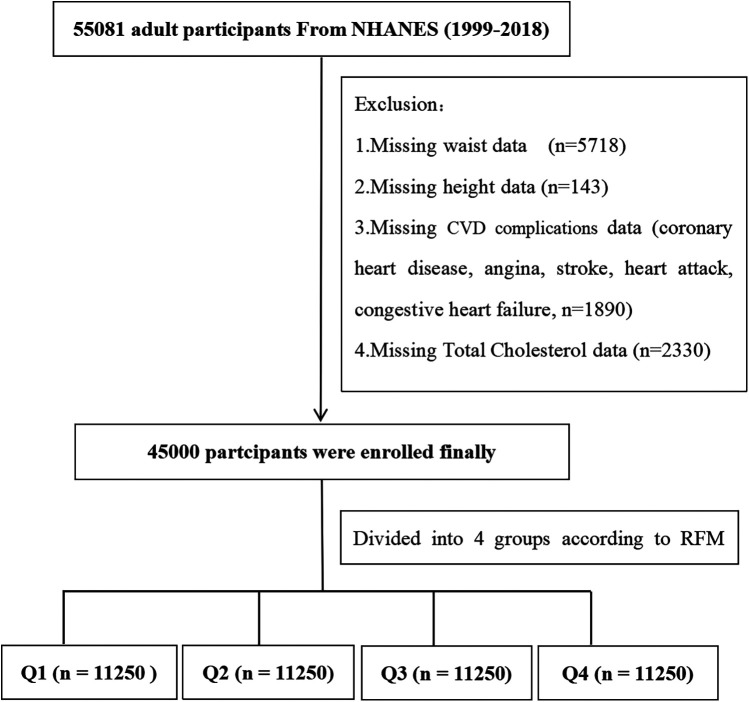
Flow diagram of the screening and enrollment of study participants.

Figure 1 illustrates the flow of participants through the study. An initial sample size of 55,081 adult participants. Exclusions were made for unavailable waist circumference data (*n* = 5,718), height data (*n* = 143), CVD complications data (*n* = 1,890), and TC data (*n* = 2,330). Ultimately, 45,000 participants were enrolled. They were further divided into quartiles based on their RFM index: Q1 (*n* = 11,250), Q2 (*n* = 11,250), Q3 (*n* = 11,250), and Q4 (*n* = 11,250).

### Baseline characteristics

The characteristics of the study group are outlined in Table 1, categorized by RFM quartiles. The average age recorded among the 45,000 participants was 49.66 ± 17.78 years, and the prevalence of CVD was 10.4%. Those grouped in the upper RFM quartiles (Q4) tended to be older, with greater BMI and waist circumference, and exhibited higher prevalence of hypertension and diabetes.

**Table 1:** Baseline characteristics of the study population according to the quartiles of the RFM.

Variables	Total (*n* = 45,000)	Q1 (*n* = 11,250)	Q2 (*n* = 11,250)	Q3 (*n* = 11,250)	Q4 (*n* = 11,250)
Age (years)	49.66 ± 17.78	44.24 ± 17.53	51.29 ± 17.79	49.72 ± 17.62	53.39 ± 16.86
Gender (%)					
Male	22,371 (49.71)	10,862 (96.55)	8,892 (79.04)	2,597 (23.08)	20 (0.18)
Female	22,629 (50.29)	388 (3.45)	2,358 (20.96)	8,653 (76.92)	11,230 (99.82)
Race, *n* (%)					
1	20,101 (44.67)	4,943 (43.94)	5,446 (48.41)	5,266 (46.81)	4,446 (39.52)
2	9,091 (20.21)	2,594 (23.06)	1,807 (16.06)	2,046 (18.19)	2,644 (23.51)
3	7,931 (17.62)	1,566 (13.92)	2,128 (18.92)	1,829 (16.26)	2,408 (21.40)
4	3,777 (8.39)	753 (6.69)	931 (8.28)	998 (8.87)	1,095 (9.73)
5	4,100 (9.11)	1,394 (12.39)	938 (8.34)	1,111 (9.88)	657 (5.84)
Weight (kg)	81.06 ± 20.73	74.52 ± 12.73	83.66 ± 19.27	77.86 ± 25.88	88.19 ± 20.12
Height (cm)	167.31 ± 10.17	174.85 ± 7.95	171.13 ± 8.53	163.89 ± 8.90	159.36 ± 7.21
BMI (kg/m^2^)	28.87 ± 6.60	24.27 ± 3.11	28.25 ± 4.72	28.45 ± 6.76	34.53 ± 6.55
Waist (cm)	98.71 ± 16.01	88.46 ± 9.08	100.14 ± 14.45	96.86 ± 18.69	109.37 ± 12.66
RFM	35.32 ± 8.60	24.51 ± 3.73	31.68 ± 1.59	38.54 ± 2.30	46.54 ± 2.80
Marry, *n* (%)					
1	27,090 (60.76)	6,792 (61.00)	7,673 (68.8)	6,758 (60.63)	5,867 (52.60)
2	17,497 (39.24)	4,342 (39.00)	3,479 (31.2)	4,388 (39.37)	5,288 (47.40)
PIR, *n* (%)					
1	12,497 (30.32)	3,010 (29.08)	2,741 (26.58)	2,937 (28.42)	3,809 (37.30)
2	15,682 (38.05)	3,777 (36.49)	3,904 (37.85)	3,984 (38.55)	4,017 (39.33)
3	13,035 (31.63)	3,565 (34.44)	3,669 (35.57)	3,414 (33.03)	2,387 (23.37)
Education, *n* (%)					
1	11,888 (26.44)	2,747 (24.44)	3,002 (26.71)	2,647 (23.56)	3,492 (31.07)
2	10,399 (23.13)	2,579 (22.94)	2,544 (22.63)	2,562 (22.8)	2,714 (24.15)
3	22,668 (50.42)	5,914 (52.62)	5,694 (50.66)	6,027 (53.64)	5,033 (44.78)
Smoking status, *n* (%)					
Never	24,301 (54.04)	5,329 (47.41)	5,219 (46.43)	6,695 (59.54)	7,058 (62.79)
Former	11,113 (24.71)	2,554 (22.72)	3,692 (32.84)	2,513 (22.35)	2,354 (20.94)
Current	9,552 (21.24)	3,357 (29.87)	2,330 (20.73)	2,036 (18.11)	1,829 (16.27)
Alcohol, *n* (%)					
Never	5,783 (14.02)	863 (8.31)	886 (8.48)	1,679 (16.42)	2,355 (23.13)
Former	7,084 (17.18)	1,418 (13.65)	1,913 (18.32)	1,660 (16.23)	2,093 (20.56)
Current	28,374 (68.80)	8,108 (78.04)	7,644 (73.20)	6,889 (67.35)	5,733 (56.31)
Coronary heart disease, *n* (%)					
No	43,194 (95.99)	10,913 (97.00)	10,589 (94.12)	10,817 (96.15)	10,875 (96.67)
Yes	1,806 (4.01)	337 (3.00)	661 (5.88)	433 (3.85)	375 (3.33)
Stroke, *n* (%)					
No	43,428 (96.51)	11,001 (97.79)	10,834 (96.3)	10,875 (96.67)	10,718 (95.27)
Yes	1,572 (3.49)	249 (2.21)	416 (3.70)	375 (3.33)	532 (4.73)
Angina, *n* (%)					
No	43,753 (97.23)	11,071 (98.41)	10,872 (96.64)	10,957 (97.4)	10,853 (96.47)
Yes	1,247 (2.77)	179 (1.59)	378 (3.36)	293 (2.60)	397 (3.53)
Heart attack, *n* (%)					
No	43,126 (95.84)	10,899 (96.88)	10,591 (94.14)	10,806 (96.05)	10,830 (96.27)
Yes	1,874 (4.16)	351 (3.12)	659 (5.86)	444 (3.95)	420 (3.73)
Congestive heart failure, *n* (%)					
No	43,697 (97.10)	11,074 (98.44)	10,860 (96.53)	10,917 (97.04)	10,846 (96.41)
Yes	1,303 (2.90)	176 (1.56)	390 (3.47)	333 (2.96)	404 (3.59)
Hypertension, *n* (%)					
No	26,193 (58.21)	8,132 (72.28)	6,344 (56.39)	6,725 (59.78)	4,992 (44.37)
Yes	18,807 (41.79)	3,118 (27.72)	4,906 (43.61)	4,525 (40.22)	6,258 (55.63)
Diabetes, *n* (%)					
No	37,294 (82.88)	10,308 (91.63)	9,188 (81.67)	9,516 (84.59)	8,282 (73.62)
Yes	7,706 (17.12)	942 (8.37)	2,062 (18.33)	1,734 (15.41)	2,968 (26.38)
Cardiovascular Disease, *n* (%)					
No	40,309 (89.58)	10,464 (93.01)	9,816 (87.25)	10,152 (90.24)	9,877 (87.80)
Yes	4,691 (10.42)	786 (6.99)	1,434 (12.75)	1,098 (9.76)	1,373 (12.20)
Total cholesterol(mg/dl)	195.59 ± 42.09	190.32 ± 41.56	195.27 ± 42.44	196.52 ± 41.96	200.26 ± 41.80

Data are presented as number and percentage for categorical variables. Continuous variables were presented as mean ± SD. BMI, body mass index; RFM, relative fat mass; PIR, poverty income ratio. Race: 1-Non-Hispanic White, 2-Non-Hispanic Black, 3-Mexican American, 4-Other Hispanic, 5-Other Race—Including Multi-Racial. Marry: 1-Married Living with partner, 2-Never married/Other: widowed, divorced, or separated individuals. PIR: 1–≤1.30, 2–1.31–3.50, 3–>3.50. Education:1-Less than high school: Less than 9th Grade and 9–11th Grade (Includes 12th grade with no diploma), 2-High school or equivalent: High school graduate/GED or equivalent, 3-Above high school: Some College or AA degree and College graduate or above. Smoking and alcohol Status: 1 for never, 2 for former, and 3 for current.

### Association between RFM and CVD

Table 2 shows the results of the multivariate logistic regression analysis assessing the association between RFM and CVD. In the fully adjusted Model 3, for each unit increase in RFM, the odds of developing CVD rose by 4% (OR = 1.04; 95% CI: 1.03–1.05; *P* < 0.001). Individuals in the highest RFM quartile (Q4) exhibited a significantly higher risk of CVD, with a 2.11-fold increase compared to those in the lowest quartile (Q1) (OR = 2.11; 95% CI: 1.76–2.53; *P* < 0.001). A significant trend was observed across the quartiles (*P* < 0.001), suggesting a dose-dependent relationship between RFM and the likelihood of CVD.

**Table 2:** Association between RFM and CVD.

RFM	n.total	n.event_%	Model1		Model2		Model3	
			OR (95%CI)	*P*-value	OR (95%CI)	*P*-value	OR (95%CI)	*P*-value
Continuous	45,000	4,691 (10.42)	1.02 (1.02–1.02)	<0.001	1.07 (1.06–1.08)	<0.001	1.04 (1.03–1.05)	<0.001
Quartile								
Q1 (6.750–7.887)	11,250	786 (6.98)	1(Ref)		1(Ref)		1(Ref)	
Q2 (7.887–8.216)	11,250	1,434 (12.75)	1.94 (1.78–2.13)	<0.001	1.53 (1.38–1.68)	<0.001	1.33 (1.19–1.48)	<0.001
Q3 (8.216–8.627)	11,250	1,098 (9.76)	1.44 (1.31–1.58)	<0.001	2.34 (2.07–2.65)	<0.001	1.62 (1.40–1.86)	<0.001
Q4 (8.627–12.481)	11,250	1,373 (12.20)	1.85 (1.69–2.03)	<0.001	4.03 (3.46–4.71)	<0.001	2.11 (1.76–2.53)	<0.001
*P* for trend	45,000	4,691 (10.42)	1.15 (1.12–1.18)	<0.001	1.58 (1.50–1.66)	<0.001	1.28 (1.21–1.36)	<0.001

Model1, unadjusted model; Model2, adjusted for age, gender, race; Model3, adjusted for model2 + Marry, PIR, Education, smoke, alcohol, hypertension, diabetes, and total cholesterol.

**Figure 2:**
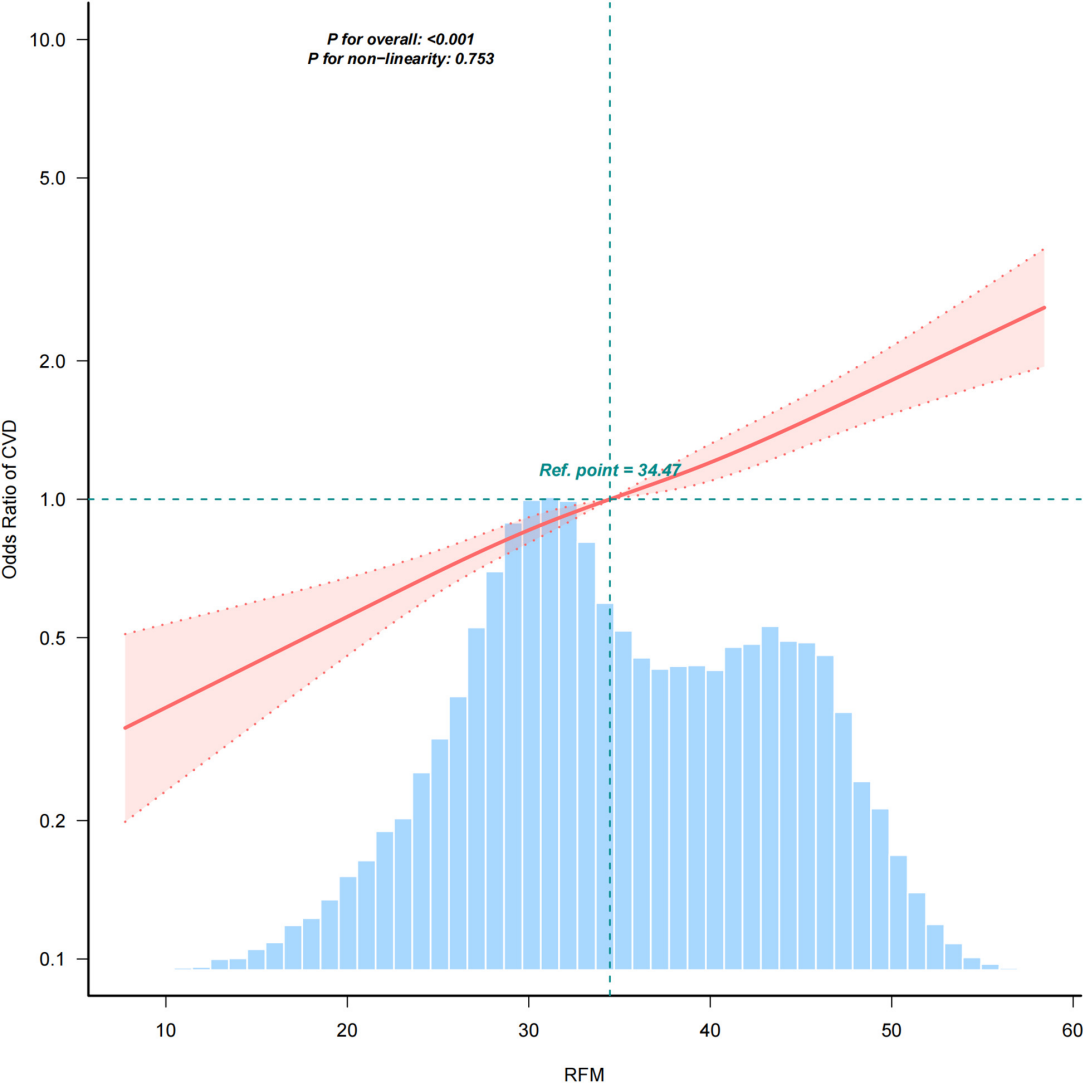
Positive relationship between RFM and CVD. Adjusted for age, gender, race, Marry, PIR, Education, smoke, alcohol, hypertension, diabetes, and total cholesterol.

Figure 2 presents linear relationship between RFM and CVD risk using a smooth curve fitting approach. The OR of CVD increased progressively with higher RFM values. The overall *P* value was <0.001, indicating a significant association, while the *P* value for non-linearity was 0.753, suggesting a linear relationship between RFM and CVD risk.

### Subgroup and interaction analyses

**Figure 3:**
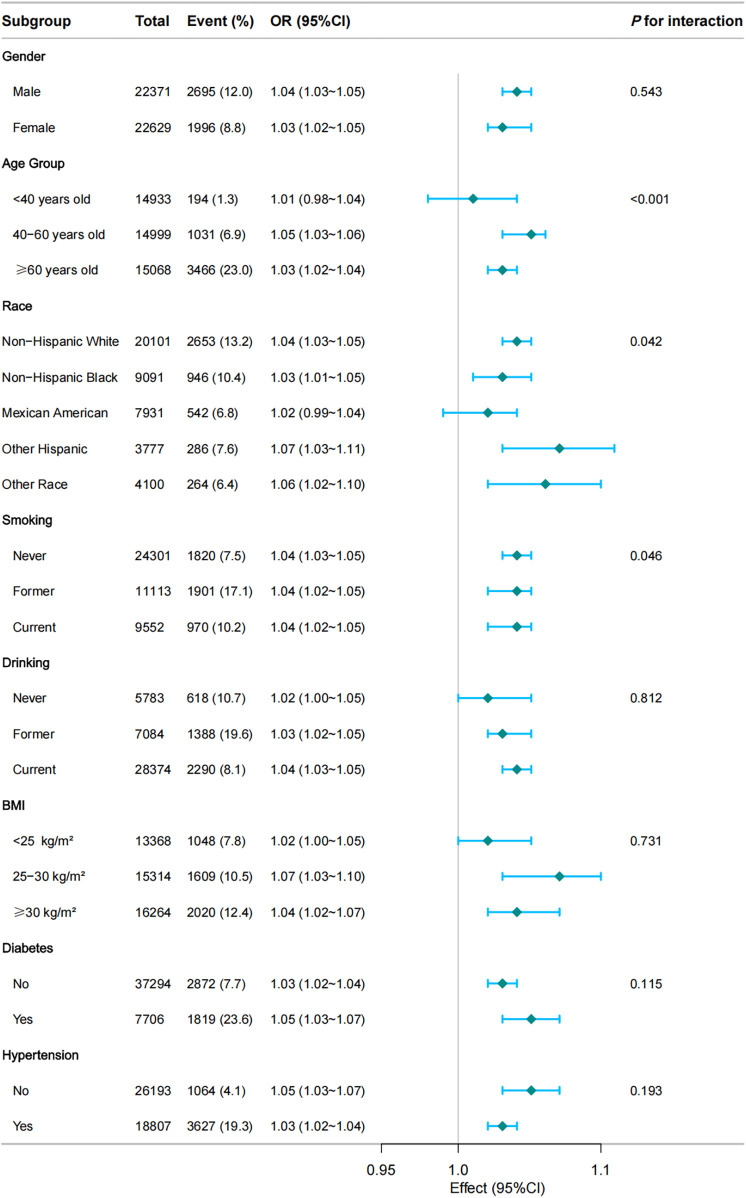
Subgroup and interaction analyses of the RFM and CVD. Multivariable logistic model adjusted for age, gender, race, Marry, PIR, Education, smoke, alcohol, hypertension, diabetes, and total cholesterol.

Figure 3 displays the results of subgroup and interaction analyses. The association between RFM and CVD was stronger in certain subgroups, including individuals aged <60 years (OR = 1.05; 95% CI: 1.03–1.06), non-Hispanic Whites (OR = 1.04; 95% CI: 1.03–1.05), and those with BMI <30 kg/m^2^ (OR = 1.07; 95% CI: 1.03–1.11). No significant interactions were observed for gender (*P* = 0.543) or hypertension (*P* = 0.193), indicating that the association between RFM and CVD was consistent across these factors.

### Sensitivity analyses

In Table 3, the results of sensitivity analyses performed among the non-Hispanic White population (*n* = 20,101) are detailed. The correlation between RFM and CVD persisted as significant (OR = 1.04; 95% CI: 1.03–1.05; *P* < 0.001), with similar trends observed across RFM quartiles. These outcomes underscore the reliability of the primary analysis and indicate that the observed association is not attributable to particular racial or ethnic groups.

**Table 3:** Sensitivity analyses: association between RFM and CVD.

RFM	n.total	n.event_%	Model 1		Model 2		Model 3	
			OR (95%CI)	*P*-value	OR (95%CI)	*P*-value	OR (95%CI)	*P*-value
Continuous	20,101	2,653 (13.20)	1.02 (1.02–1.03)	<0.001	1.07 (1.06–1.08)	<0.001	1.04 (1.03–1.05)	<0.001
Quartile								
Q1 (6.750 −7.887)	4,943	438 (8.86)	1 (Ref)		1 (Ref)		1 (Ref)	
Q2 (7.887–8.216)	5,446	866 (15.90)	1.94 (1.72–2.20)	<0.001	1.49 (1.30–1.70)	<0.001	1.31 (1.13–1.52)	<0.001
Q3 (8.216–8.627)	5,266	670 (12.72)	1.50 (1.32–1.70)	<0.001	2.36 (2.01–2.78)	<0.001	1.64 (1.37–1.98)	<0.001
Q4 (8.627–12.481)	4,446	679 (15.27)	1.85 (1.63–2.11)	<0.001	3.97 (3.23–4.87)	<0.001	2.09 (1.64–2.65)	<0.001
*P* for trend	20,101	2,653 (13.20)	1.15 (1.10–1.19)	<0.001	1.57 (1.47–1.68)	<0.001	1.28 (1.18–1.38)	<0.001

Model1, unadjusted model; Model2, adjusted for age, gender, race; Model3, adjusted for model2 + Marry, PIR, Education, smoke, alcohol, hypertension, diabetes, and total cholesterol.

## Discussion

Our results demonstrated a significant mild positive association between RFM and CVD risk. The relationship between the variables remained robust even after accounting for a range of covariates, such as sex, age, ethnicity, marital status, PIR, education level, smoking status, alcohol consumption, hypertension, diabetes, and TC. Fully adjusted models showed a significant positive association between RFM and CVD (OR = 1.04; 95% CI = 1.03–1.05; *P* < 0.001), with participants in the highest RFM quartile having a 2.11-fold increased risk of CVD compared to those in the lowest quartile (OR = 2.11; 95% CI: 1.76–2.53; *P* < 0.001). A clear linear dose-response pattern was observed, with a significant trend evident across the quartiles of RFM (*P* for trend <0.001). Further subgroup analyses indicated that this association was more pronounced among individuals under 60 years of age, non-Hispanic White participants, and those with a BMI below 30 kg/m^2^.

The results obtained align with earlier researches that had investigated the connection between various obesity indicators and the risk of CVD. For example, a study by Wang et al. (14) found that elevated RFM was associated with increased cardiovascular mortality, highlighting the importance of RFM as a predictor of cardiovascular outcomes. Similarly, Peng et al. (11) demonstrated that RFM could accurately predict hypertension, a key precursor to CVD. However, our study extends these findings by using a large, nationally representative data set and adjusting for a comprehensive set of covariates. Notably, A comprehensive analysis found a weaker association between BMI and CVD risk compared to RFM. This discrepancy may be attributed to the fact that RFM provides a more accurate assessment of body fat percentage compared to BMI, which does not differentiate between fat and muscle mass (10). Additionally, our study focused on a broader range of cardiovascular outcomes (coronary heart disease, stroke, angina, heart attack, or been diagnosed with congestive heart failure), whereas some previous studies have concentrated on specific conditions such as stroke (13).

The observed linear relationship between RFM and CVD risk can be attributed to several underlying biological mechanisms. Elevated RFM is indicative of increased total body fat, which is associated with metabolic dysregulation, inflammation, and endothelial dysfunction—all critical pathways in the development of CVD (15–18). Excess adipose tissue releases pro-inflammatory cytokines, which promote chronic low-grade inflammation and contribute to atherosclerosis (19). Additionally, increased adiposity impairs insulin sensitivity, leading to hyperglycemia and dyslipidemia, both of which are major risk factors for CVD (20). Furthermore, adipose tissue dysfunction can lead to elevated levels of free fatty acids and a decrease in the availability of nitric oxide, resulting in impaired endothelial function and heightened vascular resistance (21–24). These factors together elevate the likelihood of CVD in individuals presenting with a higher RFM. Nonetheless, it should be acknowledged that these biological processes serve as potential explanations, yet they remain unverified within the context of this study. Due to the cross-sectional design of the research, it is not possible to establish definitive causal links or confirm the mechanisms that connect RFM to CVD. Future investigations need to prioritize longitudinal studies and experimental methodologies to clarify the causal relationships and the underlying dynamics involved (25). Additionally, further investigation into potential interventions targeting RFM reduction and their impact on cardiovascular health is warranted. This could include lifestyle interventions, pharmacological treatments, or public health initiatives aimed at reducing obesity and its associated cardiovascular risks.

### Strengths and limitations

This research boasts numerous advantages. To begin with, leveraging NHANES data guarantees a substantial and representative sample across the USA nation, thereby bolstering the broader applicability of the results. Secondly, a thorough array of confounding factors was incorporated in the analysis, allowing for strong adjustments and minimizing the potential for bias. In a further step, the study applied a specific method known as RCS regression to assess the relationship between different levels of RFM and the incidence of CVD, which enhanced the insight into this association. Nevertheless, certain constraints are present. To begin with, the cross-sectional nature of the study inhibits the ability to infer causality and prevents the evaluation of relationships over time. Additionally, the findings of our research were based on data provided by participants regarding their cardiovascular disease status, which could lead to potential biases related to memory recall. Moreover, even after accounting for various confounding factors, there remains the possibility of residual confounding arising from unassessed elements such as levels of physical activity and dietary habits.

## Conclusion

Elevated RFM is associated with an higher proportion of patients with CVD, highlighting the potential benefits of managing RFM for CVD prevention. Further research is warranted to explore the underlying mechanisms and validate the use of RFM as a clinical tool for CVD risk assessment.

## Data Availability

Publicly available datasets were analyzed in this study. This data can be found here: https://www.cdc.gov/nchs/nhanes/.
