# Supplementary material for: Association between relative fat mass and cardiovascular disease: a cross sectional study based on NHANES
Source: Front Cardiovasc Med. 2025 Jun 24;12:1590979. doi: 10.3389/fcvm.2025.1590979 (PMC12234565; doi:10.3389/fcvm.2025.1590979)
Supplement: Supplementary file 1 [file Table1.docx]

**Race** was categorized as: 1 represented for Non-Hispanic White, 2 for Non-Hispanic Black, 3 for Mexican American, 4 for Other Hispanic, 5 for Other Race (including multi-racial).

**Marital status** was defined as: 1 for Married or Living with partner, 2 for Never married or Other (widowed, divorced, or separated).

**PIR** was grouped as: 1 for PIR ≤1.30, 2 for 1.31–3.50, 3 for >3.50. Education level was categorized as: 1 for Less than high school (less than 9th grade and 9–11th grade, including 12th grade with no diploma), 2 for High school or equivalent (high school graduate/GED), 3 for Above high school (some college or AA degree, college graduate or above).

**Smoking Status**: Defined as never (smoked <100 cigarettes in life), former (smoked ≥100 cigarettes in life but not currently smoking), and current (smoking some days or every day).

**Drinking Status**: Defined as never (consumed <12 drinks in lifetime), former (consumed ≥12 drinks in 1 year but not in the past year, or ≥12 drinks in lifetime but not in the past year), and current (including heavy, moderate, and mild alcohol use). Current heavy alcohol use was defined as ≥3 drinks per day for females, ≥4 drinks per day for males, or binge drinking (≥4 drinks on the same occasion for females, ≥5 drinks for males) on ≥5 days per month. Current moderate alcohol use was defined as ≥2 drinks per day for females, ≥3 drinks per day for males, or binge drinking ≥2 days per month. Current mild alcohol use was defined as ≤1 drink per day for females, ≤2 drinks per day for males.
